# Swain—Surgical Emergencies

**Published:** 1874-11

**Authors:** 


Surgical Emergencies: together with the emergencies attendant on parturition and the treatment of poisoning. A manual for the use of general practitioners. By William Paul Swain, F.R.C.S., Surgeon to the Royal Albert Hospital, Davenport, with 82 illustrations. Philadelphia: Lindsay & Blakiston, 1874. 12 mo., cloth, pp. 189. Price $2.00. Sold in Atlanta, by J. J. & S. P. Richards.

In striking contrast with the more pretentious work of Dr. Clay, of Manchester, we find in the title-page and preface of the hand-book of Mr. Swain, of Davenport, an exhibition of scholarly modesty and frankness which serves as a fitting prelude to the freshness and general excellency of its contents. At the very outset the author informs us: "This manual pretends to be little more than a compilation from the best and most recent works on surgery. It is intended to supply what the author believes is a wide-spread want, viz: a small book containing directions for the immediate treatment of all those various emergencies with which the general practitioner may be called upon to deal at any moment. The aim has been throughout to condense as much as possible, without sacrificing precision and clearness in detail." A ready remembrancer, to suggest at the moment what one needs in a large variety of surgical emergencies, when there is not time for more leisure and more satisfactory study, is what Mr. Swain would give us; and in the execution of this purpose we think he has been happily successful.

We are not of those who would decry such hand-books—such superficial and easy paths to useful knowledge. While we would freely admit that they have an alluring tendency to discourage the proper study of more ponderous, more complete, and more scientific treatises, still we must recollect that the mass of medical men are not, nor can they be, at present, erudite scholars. If, by the use of these short routes, the less learned of our profession can be imbued with increased stores of practical information bearing upon their daily duties, the public is thereby greatly benefited, and the more learned of the profession ought not to complain. It does not follow that the acquisition of such superficial knowledge by the great mass of practitioners will tend to destroy, or even to depreciate, the real value of erudition, or, indeed, its purchasing power in the markets of the world. It should be borne in mind that, while the ground floor of the temple of science may be inconveniently crowded, there is always abundant room in the upper story, and ample inducements for those who have capacity to climb there.

